# Topographical diversity of common skin microflora and its association with skin environment type: An observational study in Chinese women

**DOI:** 10.1038/s41598-017-18181-5

**Published:** 2017-12-22

**Authors:** Xi Li, Chao Yuan, Licong Xing, Philippe Humbert

**Affiliations:** 1 Clinical Research APAC, Johnson & Johnson (China) Ltd., Worldwide EM Innovation Center, 3285 Dongchuan Road, Minhang District, Shanghai 200245 China; 2 Skin & Cosmetic Research Department, Shanghai Skin Disease Hospital, 1278 Bao De Road, Jing’an District, Shanghai 200443 China; 3 Department of Dermatology, Research and Clinic Centre on the Tegument, Clinical Investigation Center, Besançon University Hospital, Besançon, France; 4 University of Franche-Comté, Inserm, U1098 Besançon, France

## Abstract

This study evaluated cutaneous microbial distribution, and microbial co-occurrence at different body sites and skin environments in Chinese women (39.6 ± 11.9 years, N = 100) during the winter season. Microbial distribution (*Propionibacterium acnes*, *Staphylococcus aureus*, *Staphylococcus epidermidis*, *Lactobacillus*, Pseudomonadaceae, and *Malassezia furfur*), association with biomarkers (antimicrobial peptides: LL-37, β-defensins [HBD-2, HBD-3]), and claudin-1) and skin biophysical parameters (transepidermal water loss, pH, skin scaliness and roughness, sebum and hydration levels) were also determined. Skin sites (glabella [GL], hand-back [HB], interdigital web-space [IS], antecubital fossa [AF], volar forearm [VF], back [BA]) were classified as normal, oily or dry based on two-step cluster analysis and exposed or unexposed (uncovered or covered by clothes, respectively) based on seasonal apparel. Pseudomonadaceae and *Staphylococcus aureus* had the highest and lowest detection rate respectively at all sites. Cluster analysis identified skin sites as ‘normal’ (HB, BA, AF, VF), ‘dry’ (IS) and ‘oily’ (GL). Bacterial alpha diversity was higher in exposed (HB, IS, and GL) compared with unexposed sites (BA, AF and VF). Co-occurrence of *Staphylococcus aureus* with any of the other five microorganisms was lower in dry and oily skin versus normal skin. Skin exposure, biophysical/barrier profile and biomarkers were found to be associated with bacterial distribution and co-occurrence.

## Introduction

The intricate structure of the cutaneous system represents an ecosystem harbouring a multitude of microorganisms including bacteria, fungi, viruses, and mites, collectively referred to as the human microbiome^1–4^. These microflora are in equilibrium with the host innate immune system and maintain homeostasis, which when altered directly impacts skin health. Skin microbial imbalance or a shift in the abundance of resident microbial taxa may be a determining factor in various disorders such as acne, atopic dermatitis, and psoriasis^4–6^.

The skin is a critical barrier between the body and the external milieu comprising a “physical barrier” (environment, surface pH, lower temperature, acidic nature, timely desquamation, and tight junction proteins) and a “chemical barrier” (host defence molecules released by keratinocytes such as anti-microbial peptides [AMPs] [e.g. defensins, cathelicidin LL-37, and dermcidin], cytokines, proteases, lysozymes, and chemokines)^1,7–9^. These barriers safeguard against pathogen invasion and colonization^3,4,10^. The human microbiome is dynamic and exhibits diversity within and across individuals, which is attributable to genetic and demographic properties, age, gender, ethnicity, skin type, lifestyle, hygiene, geographical differences, environmental stress (temperature, moisture, seasonal variation, radiation exposure) and cohabitation with other animals^4,11–15^. Studies have also suggested that biophysical parameters (such as surface pH, hydration, sebum content, transepidermal water loss [TEWL], and barrier function) vary with the age, gender, and body site, which in turn influence microbial composition^16–18^. Bacterial colonization relies on the physiology of skin and is influenced by invaginations, appendages, and the skin micro-environment (e.g., humid, dry or sebaceous, exposure to macro environment) and has an impact on skin health^4,6,18,19^. Also, commensal microbes, which prevent colonization of opportunistic or pathogenic organisms, produce AMPs and play a critical role in modulating both innate and adaptive immune response^1^.

Comprehensive understanding of the topographical and temporal diversity of the skin microbiome and associated biophysical parameters may unveil the relationship between skin health and disorders^5^. Furthermore, it can also aid in understanding subclinical skin changes, which may help in identifying the role of prebiotics and/or probiotics in skin disorders (e.g. acne), wound healing, and photoprotection^5,20–24^. In addition, dermatological treatment should be tailored to population-specific approach, avoiding extrapolation from global studies or from dissimilar populations^25^.

Previous studies have reported that the skin of Chinese populations has distinct microbiomes and Actinobacteria (*Propionibacterium*, *Corynebacterium* and *Micrococcus*), Firmicutes (*Staphylococcus* and *Lactobacillus*), Proteobacteria (Pseudomonadaceae), and fungi (*Malassezia*) as commonly occurring microbial phyla with varying relative abundance at different skin sites^13,15,26,27^. These earlier studies reported the overall distribution of the microbiome at different skin sites, without demonstrating a clear association with skin physiology. In contrast, the present study focuses on microbial co-occurrence and association with sub-clinical skin physiology and biomarkers for commonly observed skin microflora in Chinese women: *Propionibacterium acnes* (*P*. *acnes*), *Staphylococcus aureus* (*S*. *aureus*), *Staphylococcus epidermidis* (*S*. *epidermidis*), *Lactobacillus*, Pseudomonadaceae, and *Malassezia furfur* (*M*. *furfur*).

## Methodology

### Study participants

Healthy women between 20–60 years who resided within the city area were included in this study conducted in Shanghai Skin Disease Hospital, China from February 2012 to March 2012 (during winter season). The average high and low temperatures during the study period ranged between 8–13 °C and 1–4 °C, respectively, with ~79% relative humidity^28^. Participants who were living in Shanghai for at least five years, had not received antibiotics three months before the sampling and who were willing to avoid any other medicine during the test period were recruited. Participants were asked to select a test time-point that did not overlap with their menses. Key exclusion criteria included involvement with other clinical research in the last three months, pregnant or lactating women, presence of any skin ailments (atopic dermatitis, psoriasis and stasis eczema), scar, inflammation or tattoos, which might interfere with findings of the current study.

Before the study, participants were asked to complete a self-assessment questionnaire that included basic information, habits of life, family medical history and participant’s perception on skin concerns (itches, stinging, burning, dryness, and scaling) at the selected body sites. Included participants were instructed to bathe with only water and to avoid using any personal hygiene products during the two day wash-out period. Moreover, washing the body sites chosen for the study was not allowed for 12 h (except 4 h for hands) before sampling. Swimming in chlorinated pools, or use of hot water/sauna/tanning bed was avoided.

The study protocol was approved by the Scientific and Ethical Committee at the Shanghai Skin Disease Hospital and the study was conducted in accordance with the Declaration of Helsinki Principles. All participants provided informed consent to participate in the study.

### Study Design

This observational single-centre study evaluated cutaneous microbial distribution of six microorganisms within the family, genera or species of *P*. *acnes*, *S*. *aureus*, *S*. *epidermidis*, *Lactobacillus*, Pseudomonadaceae, and *M*. *furfur* on six selected skin sites (glabella [smooth part of the forehead above and between the eyebrows], GL; hand-back, HB; interdigital web space [membranes of skin between the fingers or toes], IS; antecubital fossa [triangular area on the anterior part of the elbow], AF; volar forearm [interior surface of the forearm], VF; back, BA) representative of different skin type (classified as normal, oily and dry) and exposure status (based on seasonal apparel). The sites were clinically assessed (skin aesthetics and dermal tolerance-related assessment grading) and evaluated for biophysical parameters TEWL, skin pH, sebum and hydration levels and surface evaluation of living skin [SELS] parameters) and biomarkers (AMPs such as: LL-37, β- defensins [HBD-2, HBD-3] and claudin-1) as detailed below.

### Dermatological assessments

Aesthetic conditions and skin tolerance of the selected six skin sites were clinically evaluated by an independent dermatologist, using a clinical grading system with a 10-point grading scale, where (where “0” means most positive e.g., perfectly moisturised and 10, most negative e.g., very dry) and by participant self-assessment (response to questionnaire). The dermatologists’ evaluations included skin tolerance or skin damage signs (i.e., scaling, dryness, redness, hemangiectasis, skin integrity, and skin lesions such as acne or spots) and aesthetic conditions or skin beauty-related characteristics (i.e., skin tone, glossiness, hydration, sagging, and smoothness). The participant’s evaluations included frequency of skin concerns (i.e., itch, sting, burning, dryness, and scaling). Both the dermatologists’ objective and participants’ subjective perception were included in this study, since both may be useful for assessing skin health and damaged skin barrier. Skin aesthetic conditions included tone, glossiness, hydration, sagging, and smoothness. Skin tolerance-related assessments included scales, dryness, redness, hemangiectasis, skin integrity, and skin lesions such as acne or spots. Clinical evaluations were conducted using 9-point scales (0 = most positive response or perfectly moisturized and 10 = most negative response or very dry).

### Evaluation of biophysical parameters

The measurement of biophysical parameters of the skin was performed using 6 different instruments (Courage and Khazaka electronic GmbH, Cologne, Germany): pH meter (measures skin pH, range: 0 to 12), skin-Glossymeter GL 200 (uses reflection to measure skin gloss range: 0 to 400 Glossmeter units), Sebumeter SM 820 (uses the difference of light intensity through a plastic strip to indicate the amount of absorbed sebum, range: 0 to 442 Sebumeter^®^ units), Corneometer CM 820 (uses the high dielectric constant of water for analysing the water-related changes in the electrical capacitance of the skin to assess epidermal hydration, range: 0 to 130 arbitrary Corneometer^®^ units), Tewameter (uses diffusion in an open chamber to assess trans epidermal water loss rate, range: 0 to 70 g/m^2^h) and Visioscan^®^ VC 98 (for qualitative and quantitative direct analysis of skin surface topography)^29,30^.

### Assessment of cutaneous microflora diversity

Sample collection was performed at 6 skin sites (3 exposed sites [uncovered by clothes during study period]: GL, HB, IS and 3 unexposed sites [covered by clothes during study period]: AF, VF, BA) from each participant (left or right sides were chosen randomly). The sampling was carried out as three replicate swabs (to concentrate the sample) of six identified body sites from each participant during a week with a 1-day interval between each sampling (Monday-Wednesday-Friday). The sampling regions were swabbed for approximately 50 swabs each time with physiological saline and the samples were pooled individually before analysis in back-and-forth motion with firm pressure in a temperature and humidity controlled environment (18–22 °C; relative humidity of 40–60%) and stored at 4 °C to avoid organism growth post-sampling. DNA was extracted following the manufacturer’s protocol (QIAamp DNA Microbiome kit 2016, Qiagen, CA, USA). To quantify the total skin bacteria and fungi, real-time quantitative polymerase chain reaction (RT-qPCR) testing was performed for all specimens by amplification of extracted DNA using specific primers and the Applied Biosystems 7000 Sequence Detection System (Foster City, CA)^15^. Primer and cycle details are given in the supplementary file (Tables S1). The purified PCR products were sequenced using a GS-FLX pyrosequencing platform with Titanium chemistry (Roche, Basel, Switzerland) following manufacturer’s the directions of the manufacturer. The detailed sequencing method is described in an earlier study^15^.

### Assessment of anti-microbial peptide (AMP) biomarkers

For evaluation of AMP biomarkers, specimens of *stratum corneum* were obtained from the skin of identified test areas of healthy participants by tape-stripping (5 times in same region and using last 4/5 tapes stored at −20 °C) with Corneofix^®^ (F 20, Courage and Khazaka, Germany). Venous blood samples were collected from all the participants for detection of claudin-1.

Tape-strippings were analysed for the presence of biomarkers of AMPs (LL-37, β- defensins [HBD-2, HBD-3]). For quantification, LL-37 and β- defensins were extracted from last 4/5 tapes using 15 mL Tris buffered saline and the extract was kept at 4 °C overnight. Next, each extract was filtered through polytetrafluoroethene (PTFE) membrane and the trapped corneocytes in the membrane filter were analysed using chemiluminescence immuno-detection method (VECTASTAIN^®^ Universal Elite ABC Kit, Vector Laboratories, CA, USA). For detection of claudin-1, venous blood samples were evaluated with an ELISA kit (Immundiagnostik Bensheim, Germany).

### Statistical analysis

**Figure 1:**
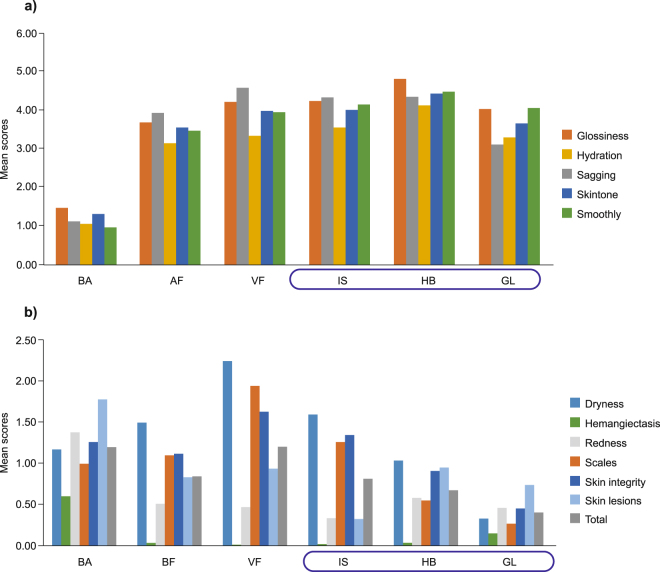
Comparison of dermal health grading of exposed and unexposed sites by dermatologist. (**a**) Skin aesthetics, and (**b**) Skin tolerance. Abbreviations: AF, antecubital fossa; BA, back; GL, glabella; HB, hand-back; IS, interdigital web space; VF, volar forearm. Boxed sites represent exposed skin sites.

Statistical analysis was performed with SPSS-17.0 (SPSS Inc., Chicago, IL, USA). Descriptive statistics by site was applied for demographic information, summarized by skin site for skin conditions, skin tolerance and aesthetics scores. Selected skin sites were differentiated as normal, dry, and oily by two-step clustering (SPSS 17.0) based on the best subsets of three skin physiology parameters [moisture, sebum, and trans-epidermal water loss (TEWL), which are considered the most representative parameters of skin micro-environment]^15,25^ as described in Fig. 1. In two-step clustering, the pre-clusters were calculated using the hierarchical clustering algorithm. Microbial diversity at different sites was calculated using alpha diversity index (Shannon and Simpson index). Paired/independent T-test and ANOVA (Dunnett’s comparison) and/or Friedman/Wilcoxon Test (for non-parametric data) were performed for each parameter to determine significant differences of subsequent readings between different sites.

Microbiome detection consistency was visualized to demonstrate microbial co-occurrence between different pairs of microorganisms. Co-occurrence between two microorganisms was defined as the percentage of sites growing both or neither of them per total number of sites. The change of co-occurrence between different skin types is represented as a matrix in a scatter diagram depicted as a point-size with different colours. Cochran–Mantel–Haenszel (CMH) test was applied for each pair of microorganisms present in the matrix to test consistency in exposed or unexposed skin sites and oily, normal or dry skin type. Change in consistency (point-size) estimates and CMH *p*-value depicts the variation in microbial co-occurrence by skin type.

The association between skin microflora, physiology, biomarkers with skin exposure status and skin type classification as dry, normal, oily was determined by regression analysis and the quantitative association was depicted by regression coefficient. To investigate association of skin microflora between exposed and non-exposed skin sites, logistic regression analysis was used to identify factors among 6 skin physiology parameters and 4 biomarkers that potentially contribute to the occurrence of each of the 6 microorganisms (indicated as positive and negative association). To investigate association of skin microflora between different skin types (normal, oily and dry), linear regression was used to identify factors (defined as any other index, biomarker or microorganism) for each skin physiology parameter. In both regression models, the identified factors were defined as P < 0.05. Based on the results from these two regression models, influence of biophysical parameters and biomarkers, occurrence of microorganisms, site groups (model-based and exposed or unexposed groups) were visualized (R package igraph) as directed social network analysis diagrams to demonstrate all relationships together, wherein P < 0.05 was considered as statistically significant.

## Results

A total of 100 Chinese women were enrolled in the study and the average age was 39.6 ± 11.9 years. All participants completed the study.

### Clinical assessment at different skin sites

**Figure 2:**
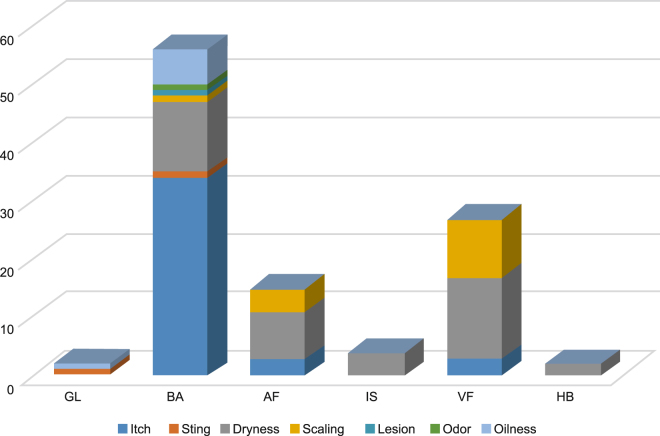
Participants perception of skin concerns at different skin sites. Abbreviations: AF, antecubital fossa; BA, back; GL, glabella; HB, hand-back; IS, interdigital web space; VF, volar forearm.

A total of 97 participants completed the questionnaire. Of these, a higher frequency of participant’s perceived skin concerns were observed at unexposed skin sites (BA [56/97; 57.73%], AF [15/97; 15.46%], and VF [27/97; 27.83%]) compared to exposed skin sites (GL [2/97; 0.02%], IS [4/97; 0.04%], and HB [2/97; 0.02%]) (Fig. 2). Clinical assessment at different skin sites showed that the measurement scores (mean ± SD) of skin aesthetic grading was lowest at unexposed BA region (1.19 ± 1.19) and the highest in exposed HB region (4.40 ± 1.45, Fig. 1a). Scores of skin tolerance grading, where lower score denotes better dermal health, were overall lower for the exposed sites (IS: 0.81 ± 0.98; HB: 0.68 ± 0.94; and GL: 0.40 ± 0.67) and higher for the unexposed sites (BA: 1.19 ± 1.19; AF: 0.84 ± 1.04; and VF: 1.20 ± 1.11) (Fig. 1b).

### Evaluation of biophysical parameters

Biophysical parameters were evaluated to ascertain skin barrier properties which may have an impact on microbial distribution. The GL had significantly (p < 0.05) higher sebum levels compared with AF, IS, VF and HB; higher *stratum corneum* hydration compared with VF and HB; and skin gloss compared with AF and IS. The mean pH of the skin was 5.3 (range: 5.26 GL to 5.63 HB) and was not significantly different among the sites. TEWL was significantly higher in IS (p < 0.05) compared with AF and VF. Skin pH, skin roughness (SEr) and skin scaliness (SEsc) showed no significant differences between the 6 selected sites (Table 1).

**Table 1:** Biophysical parameters at different skin sites (Mean ± SD).

Parameters	Instrument	GL	IS	HB	BA	AF	VF
Water content/epidermal hydration, arbitrary Corneometer^®^ units	Corneometer^a^	68.92 ± 10.71^♥^	10.21 ± 11.12^♥^	45.87 ± 12.02	53.91 ± 8.78^♥^	49.88 ± 10.48^♥^	44.02 ± 9.59
TEWL, g/m^2^ h	Tewameter^b^	10.65 ± 4.58^◊^	31.45 ± 11.22^◊^	7.54 ± 3.08^◊^	4.66 ± 2.89^◊^	2.38 ± 2.05	3.01 ± 2.45
Sebum, Sebumeter^®^ units	Sebumeter^c^	106.00 ± 75.14^♦^	4.06 ± 11.98	3.27 ± 5.84	31.24 ± 34.62^♦^	8.91 ± 21.46	12.33 ± 38.00
Gloss, glossmeter units	Glossmeter^d^	10.69 ± 2.60^▲^	4.99 ± 1.29	6.78 ± 2.42^▲^	7.33 ± 1.79^▲^	4.82 ± 0.65	8.57 ± 1.56^▲^
pH	pH meter	5.26 ± 0.46	5.36 ± 0.56	5.63 ± 0.52	5.43 ± 0.53	5.30 ± 0.43	5.56 ± 0.49
SEr	Visioscan	3.64 ± 1.29	3.76 ± 1.73	3.08 ± 1.46	2.95 ± 1.87	2.11 ± 0.82	3.49 ± 1.53
SEsc		0.87 ± 0.35	1.44 ± 0.65	0.99 ± 0.55	0.62 ± 0.15	1.04 ± 0.43	1.46 ± 0.66

^♥^P < 0.05, compared to VF and HB; ^◊^P < 0.05, compared to AF and VF; ^♦^P < 0.05, compared to AF, IS, VF and HB; ^▲^P < 0.05, compared to AF and IS.

Abbreviations: AF, antecubital fossa; BA, back; GL, glabella; HB, hand-back; IS, interdigital web space; SEr, skin roughness, SEsc, skin scaliness; TEWL, transepidermal water loss; VF, volar forearm.

Parameter description

SEsc: Scaling calculated as a portion of light pixels (gray level higher than established threshold).

SEr: Roughness calculated as a portion of dark pixels (gray level is below established threshold).

### Skin site cluster analysis and associated microflora

**Figure 3:**
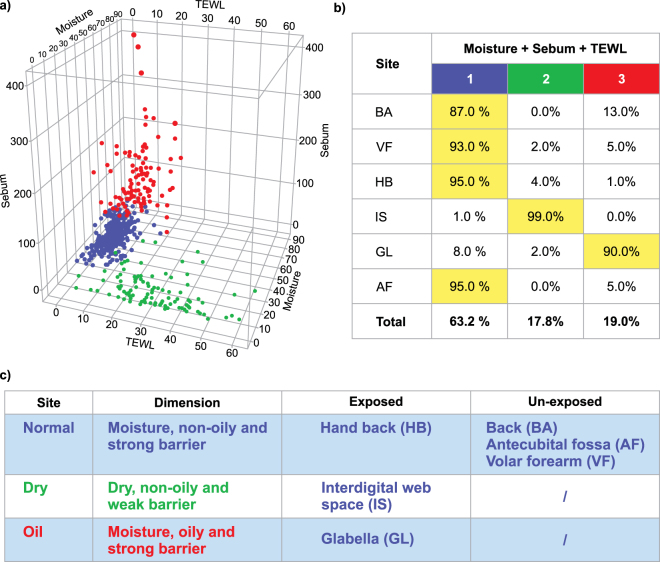
Differentiation of skin sites by cluster analysis based on moisture, sebum and transepidermal water loss. Blue, normal skin; red, oily skin; green, dry skin; skin sites (GL, HB, IS, AF, VF, BA) were classified as normal (HB, BA, AF, VF), oily (GL) or dry (IS) based on two-step cluster analysis and as exposed (HB, IS, GL) or unexposed (BA, AF, VF) based on seasonal apparel (exposed, uncovered by clothes/exposed to UV radiations during study period; unexposed, covered by clothes/ without direct UV exposure during study period). (**a**) Cluster analysis was based on three parameters (moisture, sebum and TEWL) and classified the three skin types with respect to these parameters. Coloured points representing skin types on the cluster plot denote the relative moisture, sebum or TEWL. The high TEWL values represents higher water loss and weakening of the skin barrier; higher sebum values are associated with higher oiliness of the skin. (**b**) Percentages given in the rows denote the distribution of every skin site to three model-generated skin groups among all participants. Each group (column) included some dominant skin sites (highlighted). (**c**) Considering the location and characteristic of the 6 skin sites, three model-generated skin groups are named as “Normal”, “Dry” and “Oily” skin sites. In each group, sites can also be classified into whether the site is exposed to UV radiations or covered by clothes to prevent UV radiations (un-exposed). Abbreviations: AF, antecubital fossa; BA, back; GL, glabella; HB, hand-back; IS, interdigital web space; TEWL, transepidermal water loss; VF, volar forearm.

Based on the similarity in cluster analysis (Fig. 3) of core biophysical parameters (moisture, sebum, and TEWL) and the number of samples in each cluster, skin sites were classified into 3 clusters. ‘Normal’ (cluster 1; moisture, non-oily and strong barrier) representing: HB (95%), BA (87%), AF (95%), and VF (93%) sites; ‘dry’ (cluster 2; dry, non-oily and weak barrier): IS (99%); and ‘oily’ (cluster 3; moisture, oily and strong barrier): GL (90%).

### Diversity of skin microflora at different skin sites

The occurrence of *P*. *acnes*, *S*. *aureus*, *S*. *epidermidis*, *Lactobacillus*, Pseudomonadaceae, and *M*. *furfur* species varied with different skin sites. The lowest detection rate was observed for *S*. *aureus* in all 6 sites compared with other microorganisms, whereas, the highest detection rate was observed for Pseudomonadaceae, with a highest occurrence in IS region (p < 0.05, compared with other sites, Table 2). A similar pattern was observed in the exposed as well as unexposed sites. Targeted bacterial subset alpha diversity was higher in exposed sites (HB, IS, and GL) compared with the unexposed sites (BA, AF and VF). Shannon diversity index (H) progressively decreased in the order of IS > GL > HB > AF > VF > BA with diversity index value, H = [0.88 to 1.01] for exposed sites and H = [0.64 to 0.75] for the unexposed sites. Simpson index, a measure of dominance (D) of the species increased in the order of IS < GL < HB < AF < VF < BA (Table 2, Fig. S1). As per the cluster analysis, *S*. *epidermidis* predominantly occurred in the oily and dry clusters, followed by Pseudomonadacea*e* and *P*. *acnes*, whereas Pseudomonadaceae occurrence was higher in the normal cluster (Table 2).

**Table 2:** Relative occurrence of each microorganism at different skin sites (n = 100).

Site type	Skin site	Staphylococcus aureus (n)	Staphylococcus epidermidis (n)	Lactobacillus (n)	Malassezia furfur (n)	Propionibacterium acnes (n)	Pseudomonadaceae (n)
Exposed sites	GL (oily)	4	85^#▲■^	35^#▲■^	16	83^#▲^*^◆■^	74^*■^
	IS (dry)	3	86^#▲■^	47^#▲^	26^#▲■^	70^♥▲^	87^♥#▲◆■^
	HB (normal)	3	81^#▲■^	43^#▲^	20^*^	67^♥▲^	74^*■^
Unexposed sites	BA (normal)	1	53^♥#*◆^	12^♥^	14^*^	61^♥^	60^♥*◆^
	AF (normal)	1	67^♥*◆■^	19^♥*◆^	14^*^	59^♥^	70^*^
	VF (normal)	3	55^♥*◆^	19^♥*◆^	10	48^♥*◆^	65^*^

N, total number of Chinese women enrolled; n, number of women with a specific microorganism at a particular skin site.

^♥^P < 0.05, compared to GL; ^★^P < 0.05, compared to IS; ^◆^P < 0.05, compared to HB; ^■^P < 0.05, compared to BA; ^#^P < 0.05,compared to AF; ^▲^P < 0.05, compared to VF.

Abbreviations: AF, antecubital fossa; BA, back; GL, glabella; HB, hand-back; IS, interdigital web space; VF, volar forearm.

### Evaluation of biomarkers

It was of interest to determine the abundance of AMPs, whose antibacterial properties might modulate bacterial populations, at each of the 6 skin sites. Significantly higher distribution of HBD-3 and LL-37 was observed in GL followed by BA compared with all other sites (p < 0.05). With HBD-2, a similar pattern was noted except for BA site, which did not differ significantly with GL region (Table 3).

**Table 3:** Distribution of biomarkers at different skin sites (Mean±SD).

Site type	Skin sites	HBD-2 (pg/ml)	HBD-3 (pg/ml)	LL-37 (pg/ml)	Claudin-1 (ng/ml)
Exposed sites	GL (oily)	0.27 ± 0.51^#▲*◆^	0.47 ± 0.63^#▲★◆■^	0.84 ± 0.90^#▲*◆■^	14.72 ± 6.64^♣^
	IS (dry)	0.12 ± 0.24^♥^	0.07 ± 0.09^♥■^	0.12 ± 0.12^♥■^	
	HB (normal)	0.14 ± 0.25^♥^	0.11 ± 0.18^♥■^	0.17 ± 0.22^♥■^	
Unexposed sites	BA (normal)	0.20 ± 0.37	0.22 ± 0.37^♥#▲*◆^	0.45 ± 0.53^♥#▲*◆^	
	AF (normal)	0.13 ± 0.26^♥^	0.08 ± 0.15^♥■^	0.17 ± 0.13^♥■^	
	VF (normal)	0.14 ± 0.29^♥^	0.08 ± 0.11^♥■^	0.17 ± 0.15^♥■^	

^♥^P < 0.05, compared to GL; ^★^P < 0.05, compared to IS; ^◆^P < 0.05, compared to HB; ^■^P < 0.05, compared to BA; ^#^P < 0.05, compared to AF; ^▲^P < 0.05, compared to VF

Abbreviations: AF, antecubital fossa; BA, back; GL, glabella; HB, hand-back; IS, interdigital web space; VF, volar forearm.

^♣^Claudin-1 values were obtained from blood sample and hence, the values are not site-specific.

### Association between skin microflora, AMP biomarkers and skin physiological microenvironment

#### Site exposure status (exposed or unexposed)

**Figure 4:**
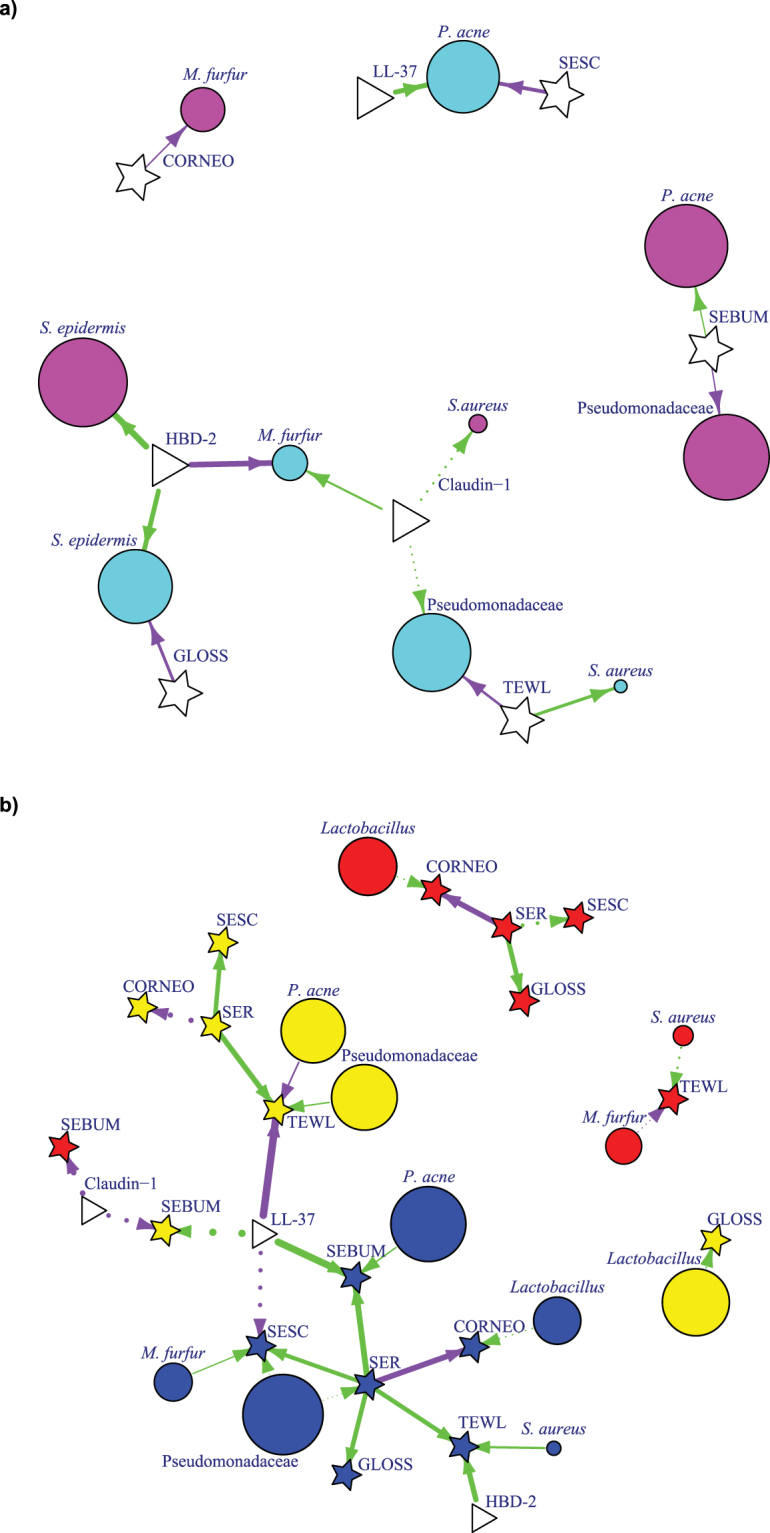
Network analysis (**a**) Association between site exposure dependent skin microflora distribution, biophysical parameters and biomarkers (by logistic regression); (**b**) Physiology dependent with other physiology parameters, microflora, and biomarkers in every skin type (by linear regression). Each node represents bacteria/fungi, the colour of the nodes corresponds to the unexposed site (cyan), exposed site (pink), normal skin site (dark blue), oil skin site (red), and dry skin site (yellow). Bacteria/microorganism are represented as circle. The size of the node corresponds to the square root of (positive rate) *30. The colour of the edges corresponds to the positive (green) or negative (purple) regression estimated coefficient. The length of the edges has no meaning. Solid line represents p < 0.05 and dotted line represents 0.05 < p < 0.1 Star shape represents biophysical parameters and skin texture index, triangles correspond to biomarkers. The width of arrow between any two elements (circle, triangle and star) represents the quantitative contribution from one element to another element, calculated based on linear regression and logistic regression model. Only significantly contributing elements were included in the figure. Abbreviations: Lactob, Lactobacillus; Malass, *Malassezia furfur*, SEsc, scaliness; SEr, roughness; TEWL, transepidermal water loss.

In exposed sites, sebum secretion appeared to correlate with *P*. *acnes* occurrence but not Pseudomonadaceae (Fig. 4a). Also, *M*. *furfur* occurrence in the exposed region was associated with less hydrated skin. In unexposed sites, occurrence of *P*. *acnes* was prominent in skin sites with less scaling and higher levels of AMP LL-37. Also, the occurrence of *S*. *epidermidis* was associated with less skin glossiness. Higher TEWL in unexposed sites was correlated with occurrence of *S*. *aureus*; however, lower TEWL was associated with occurrence of Pseudomonadaceae. A higher level of claudin-1 biomarker was associated with higher occurrence of *M*. *furfur* in unexposed sites. In addition, AMP HBD-2 did not appear to prevent the occurrence of *S*. *epidermidis* in both exposed and unexposed sites; however, a negative association was observed with the growth of *M*. *furfur* in unexposed sites.

#### Skin type (normal, oily and dry)

Higher TEWL was associated with the presence of *S*. *aureus* in normal sites and with Pseudomonadacea*e* in dry sites, whereas lower TEWL was associated with *P*. *acnes* in dry sites. Glossiness correlated with the growth of *Lactobacillus* in dry sites. In addition, skin scalinesss was supportive of *M*. *furfur* growth in normal sites (Fig. 4b). TEWL was positively associated with AMP HBD-2 in normal site and negatively associated with AMP LL-37 in the dry site. A higher sebum level in normal sites was associated with the presence of *P*. *acnes* and LL-37 (Fig. 4b).

### Microbial populations in different skin environment

#### Site exposure status (exposed or unexposed)

**Figure 5:**
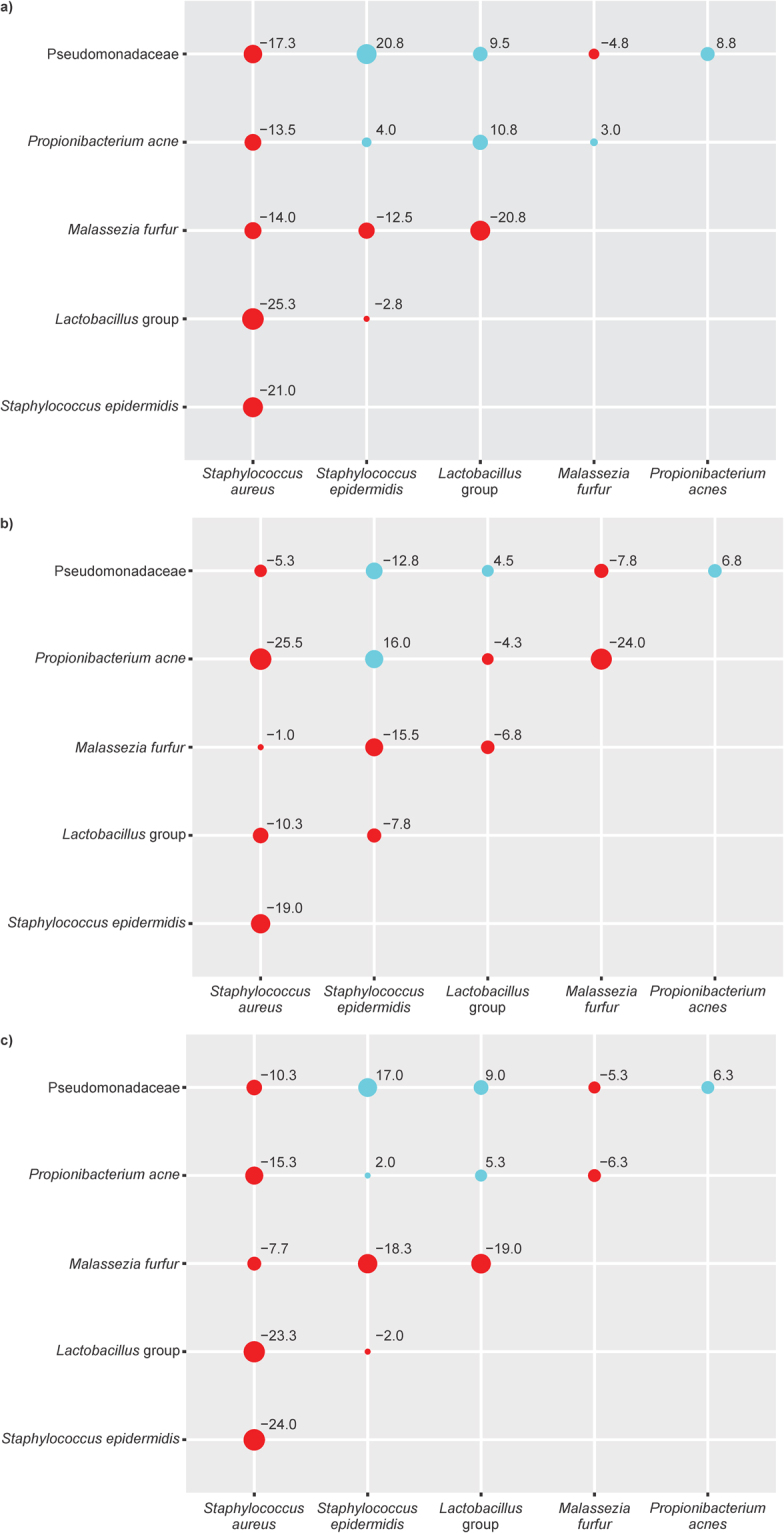
Microbial co-occurrence based on i) skin type, normal to dry (**a**), normal to oily (**b**) and ii) skin exposure (**c**). Dots represent the co-occurrence percentage difference between two given microorganisms when comparing one skin type with another, at the intersection of row and column. Red denotes decreased co-occurrence percentage compared to normal (versus dry, ‘**a**’; and versus oily ‘**b**’) and unexposed (versus exposed, ‘**c**’) sites. Green denotes increase. e.g., the co-occurrence rate between S. aureus and the five studied microorganisms decreased, while the co-occurrence rate between S. epidermis and Pseudomonadaceae increased. The more negative the value, the weaker is the co-occurrence e.g., the value −10.334 represents relatively lower co-occurrence of Pseudomonadaceae and S. aureus at exposed sites versus unexposed sites, while the value 17, represents relatively higher co-occurrence of Pseudomonadaceae and S. epidermis at exposed sites versus unexposed sites. Co-occurrence rate = (the total number of studied sites with two co-occurring microorganism + the total number of studied sites with neither of two co-occurring microorganism)/total number of studied sites.

Co-occurrence of microorganisms varied depending on skin exposure status. For instance, in exposed skin sites, co-occurrence of *S*. *aureus* with other microorganisms was lower than the unexposed sites. A similar trend was observed for *M*. *furfur*. However, co-occurrence of Pseudomonadacea*e* with *S*. *epidermidis* was higher in exposed sites compared to unexposed sites (Fig. 5a, Table S2).

#### Skin type (normal, oily and dry)

Microbial co-occurrence also differed based on the skin type. Co-occurrence of *S*. *aureus* with all the other microorganisms was lower in both dry and oily skin types as compared with normal skin. Co-occurrence of *M*. *furfur* with *Lactobacillus* in dry skin is lower in both normal, and oily skin and *P*. *acne* with *M*. *furfur* in oily skin is lower in both normal, and dry skin. However, for other microorganism pairs a clear trend was not observed. For example, co-occurrence of *S*. *epidermidis* at dry and oily sites was higher with some microorganisms (*P*. *acnes* and Pseudomonadaceae) and lower with others (*M*. *furfur* and *Lactobacillus*) as compared with normal skin type (Fig. 5b, Table S2).

## Discussion

The current study demonstrates for the first time, an association between specific skin microflora, AMP biomarkers, biophysical environment and skin types in Chinese women. The skin sites chosen belonged to distinct niches, which are known to be affected by different microorganisms associated with skin disorders such as atopic dermatitis, acne vulgaris, psoriasis etc.^4,6^.

The skin surface varies topographically and factors such as temperature, humidity, presence of sebaceous glands, exposure status influence the growth of resident microflora^4^. Therefore, each skin site exhibits specific features including location, biophysical parameters, level of exposure, and level of AMPs. Interaction of microorganisms with these parameters and with other microorganisms at the same site is potentially specific to that site. As such, six different skin sites from the same individual were selected in the current study to understand local microflora co-occurrence, its association with specific micro-environments and diversity of skin microflora. This type of analysis is potentially useful to establish healthy baselines for distinguishing between diseased and healthy skin, providing sub-clinical clues to regulate imbalanced skin at an early stage, as well as to determine the susceptibility or resistance of particular sites to therapeutics^31^. It is likely that skincare products can encompass more than one site included in this analysis. Hence, an overall picture that compares and contrasts different skin sites may be useful.

Previous studies have documented that *Propionibacterium* species mostly occur in sebaceous sites, *Staphylococcus* species in sebaceous and moist sites and a mixed population was observed in dry skin sites^5^. Earlier studies in Chinese individuals reported *Propionibacterium*, *Malassezia*, and *Staphylococcus* as commonly occurring genera in different skin sites^13,15,27^. In the current study, in all 6 skin sites monitored, *S*. *aureus* occurrence was lowest among all bacteria species examined, whereas *S*. *epidermidis* and Pseudomonadaceae were found to have high occurrences. In all skin sites classified as dry, oily or normal, *S*. *epidermidis*, Pseudomonadaceae and *P*. *acnes* predominated over other species. A significantly higher diversity of selected skin microorganisms was noted in the exposed sites compared with the unexposed regions. The Simpson index, a measure of dominance, was found to be higher for unexposed sites compared to exposed, confirming lesser diversity of specific subsets of microbiota in unexposed sites. The Shannon and Simpson indices are mainly used to measure total microbiome species diversity/abundance rather than for specific subsets of microbiota. However, in the present analysis, we used these indices to measure the abundance and diversity of six selected microorganisms; similar use of the indices for subsets has been reported in previous studies^32–35^. We consider the use of the indices to be a valid method to compare the prevalence of specific microorganisms at different skin sites. The GL, a common exposed site, showed lowest skin tolerance score but higher aesthetic grading (better dermal health) whereas BA, an unexposed site, showed highest tolerance score and lowest skin aesthetic grading score. The greater microbial diversity of exposed skin sites (compared to unexposed) is likely due to its higher interaction with the external environment, and exposure to diverse microflora^36–38^. Furthermore, exposure could also modulate resident microflora by encouraging evaporation of water, reducing the accumulation of secretions and maintaining the skin pH^18,36^. These factors may contribute to increasing the tolerability of the skin to the external environment, leading to better dermal health in exposed compared with unexposed sites. The understanding of this microbial distribution will be useful to establish a relationship between skin health and disease, which in turn may aid in the development of population-tailored treatment approaches.

Topographical variations in microbial distribution are associated with the physicochemical properties of the skin^5,31^. The present findings demonstrated that sebum-rich sites and exposed skin surfaces supported the lipophilic anaerobe *P*. *acnes* in this population of Chinese women. Similar findings were reported in earlier studies^5,15,39^. Previous studies have also reported reduced lipid production and impaired barrier function (reduced hydration and increased TEWL) in winter season. This may possibly explain the negative association between the lipophilic *P*. *acnes*, and TEWL in the dry sites during winter season in the current study^40–42^. In agreement with a study conducted in China, a positive association between *S*. *aureus* and TEWL at all sites was observed in the current study, indicating a strong relation between skin barrier impairment and *S*. *aureus* colonization^15,43^. The beneficial role of *Lactobacillus* has been confirmed in a preliminary clinical study wherein hydration and glossiness were improved, thereby delaying signs of early aging^44^. Consistent findings were noted this study, as *Lactobacillus* demonstrated positive association with hydration in oily and normal sites and glossiness in dry sites. As well, TEWL and sebum levels were observed to be negatively associated with Pseudomonadaceae whereas TEWL was positively associated with *S*. *aureus* colonization, possibly implying that the differences in the host skin physiological environment affect bacterial colonization^15,45^.

The AMPs constitute a first line of defence of the innate immune response against bacteria, viruses and fungi, and thus play a critical role in animals and humans to control the infection before the advent of symptoms^46,47^. Thus, it is necessary to consider AMPs during assessments related to skin barrier^46^. A positive association between LL-37 and *P*. *acnes* was found in the present results. An earlier study had shown increased HBD-2 and LL-37 levels along with other proinflammatory cytokines, due to the release of proteases by *P*. *acnes* ^48^. Similarly, a positive association between *S*. *epidermidis* and HBD-2 in both exposed and unexposed sites was noted in this study, which is consistent with previous studies^49,50^. Furthermore, a negative association between HBD-2 and *M*. *furfur* was noted in unexposed sites in the current study; in contrast, HBD-3 did not appear to be significantly associated with any of the microorganisms examined. The findings in this study reveal a positive association of claudin-1, a tight junction protein, with *M*. *furfur*, Pseudomonadaceae and *S*. *aureus*. Additionally, claudin-1 was found to be negatively associated with sebum at both oily and dry sites. This is consistent with earlier observations suggesting a strong link between reduced claudin-1 levels (less tight junction proteins), skin dryness and a weak barrier, which alters microflora occurrence^51,52^. Furthermore, decreased claudin-1 expression in tight junctions is found to be associated with reduced immune response and skin diseases such as atopic dermatitis and psoriasis^9,51^. Higher levels of claudin-1 in the blood can be correlated with higher occurrence of *M*. *furfur* in unexposed sites, however, as claudin-1 is present on several tissue linings, the observed higher levels in blood needs further evaluation.

Co-occurring microbes may interact with each other and compete for survival, nutrients or even immune escape mechanisms. These may either be beneficial to the host or influence the development of disease. Hence characterizing these co-occurrence patterns is an important initial step to elucidate their role in health and disease by achieving a new balanced steady state ecosystem^37^. Furthermore, with the advent of probiotic and prebiotic skin care products, association of these microbes with skin biophysical parameters, micro-environment and skin barrier function may help in designing skin care products, supporting the importance of evaluation of microbial co-occurrence at different skin sites. In the current study, overall, co-occurrence of most of the microorganism pairs was lower in the oily and dry sites as compared with the normal site. However, exceptions to this were co-occurrence of *P*. *acnes* with *S*. *epidermidis* in the oily region, *P*. *acnes* with *Lactobacillus* in the dry region, and Pseudomonadaceae with *S*. *epidermidis* in both dry and oily regions, which was higher compared with the normal sites. Additionally, with the exposure of the skin, co-occurrence of most of the microorganisms was found to be lower. Taken together, these findings suggest that co-occurrence of micro-organisms may be affected by different skin micro-environment properties, which may be due to differences in skin adaptability and barrier function. The microbial co-occurrence could also be associated with varying nutrients and metabolites provided by the different skin micro-environments of the host^53^. Other microbes residing in the skin maybe associated with skin exposure, biophysical or barrier profile and biomarkers. However, the present study only evaluates selected skin microflora as these are the most common microbial species observed at different skin sites in Chinese women^13,15,26,27^.

The present study has several limitations, including evaluation of only a subpopulation of the Chinese population (women) and their skin microflora during the winter season. Also, this study did not report the relationship between habits of life (e.g., companion animals) and skin microflora at different skin sites. Hence, the generalisation of results may have limitations owing to differences in lifestyle, diet, environmental exposure and seasonal variations. The current study is associative and an exact causative mechanism of microbial association needs further exploration. Future large studies are required to confirm current findings as well as to determine potential relationships between habits of life and skin microflora. In addition, age may have an important bearing on overall skin health, texture and barrier function, and future studies will address the influence of age on association between microorganisms, AMP biomarkers and biophysical parameters.

## Conclusion

The present findings suggest that skin exposure and skin type (as defined by sebum, hydration and physical barrier function) are important microenvironment factors that influence the targeted common microflora distribution, diversity, and co-occurrence. This underlines the importance of a comprehensive understanding of the association of microorganisms, skin biophysical parameters, microenvironment and skin barrier function including physical, chemical and microbial barriers, which is essential for designing skin care products and anti-microbial drugs. Maintaining healthy skin requires selective microbial shifts or permeability barrier changes, inhibiting the growth of pathogenic bacteria and promoting the growth of symbiotic bacteria. Hence, an alteration in the skin microflora in certain disorders by selective modulation of microflora (pre- and/or probiotics) could be a promising treatment strategy in clinical and sub-clinical skin conditions.

## Electronic supplementary material

Supplementary information

## References

[1] Christensen GJ, Bruggemann H (2014). Bacterial skin commensals and their role as host guardians. Benef. Microbes.

[2] Grice EA (2008). A diversity profile of the human skin microbiota. Genome Res..

[3] Grice EA, Segre JA (2012). Interaction of the microbiome with the innate immune response in chronic wounds. Adv. Exp. Med. Biol..

[4] Grice EA, Segre JA (2011). The skin microbiome. Nat. Rev. Microbiol..

[5] Grice EA (2009). Topographical and temporal diversity of the human skin microbiome. Science.

[6] Hannigan GD, Grice EA (2013). Microbial ecology of the skin in the era of metagenomics and molecular microbiology. Cold Spring Harb. Perspect. Med..

[7] Belizario JE, Napolitano M (2015). Human microbiomes and their roles in dysbiosis, common diseases, and novel therapeutic approaches. Front. Microbiol..

[8] Cogen AL, Nizet V, Gallo RL (2008). Skin microbiota: a source of disease or defence?. Br. J. Dermatol..

[9] Gunzel D, Yu AS (2013). Claudins and the modulation of tight junction permeability. Physiol. Rev..

[10] Proksch E, Brandner JM, Jensen JM (2008). The skin: an indispensable barrier. Exp. Dermatol..

[11] Briggs BR (2014). Seasonal patterns in microbial communities inhabiting the hot springs of Tengchong, Yunnan Province, China. Environ. Microbiol..

[12] Chen Y (2016). Changes of the Bacterial Abundance and Communities in Shallow Ice Cores from Dunde and Muztagata Glaciers, Western China. Front. Microbiol..

[13] Leung MH, Wilkins D, Lee PK (2015). Insights into the pan-microbiome: skin microbial communities of Chinese individuals differ from other racial groups. Sci. Rep..

[14] Song SJ (2013). Cohabiting family members share microbiota with one another and with their dogs. Elife.

[15] Ying S (2015). The Influence of Age and Gender on Skin-Associated Microbial Communities in Urban and Rural Human Populations. PLoS One.

[16] Man MQ (2009). Variation of skin surface pH, sebum content and stratum corneum hydration with age and gender in a large Chinese population. Skin Pharmacol. Physiol..

[17] Marrakchi S, Maibach HI (2007). Biophysical parameters of skin: map of human face, regional, and age-related differences. Contact Dermatitis.

[18] Mukherjee S (2016). Sebum and Hydration Levels in Specific Regions of Human Face Significantly Predict the Nature and Diversity of Facial Skin Microbiome. Sci. Rep..

[19] Firooz A (2012). Variation of biophysical parameters of the skin with age, gender, and body region. ScientificWorldJournal.

[20] Baquerizo Nole KL, Yim E, Keri JE (2014). Probiotics and prebiotics in dermatology. J. Am. Acad. Dermatol..

[21] Fuchs-Tarlovsky V, Marquez-Barba MF, Sriram K (2016). Probiotics in dermatologic practice. Nutrition.

[22] Grice EA (2014). The skin microbiome: potential for novel diagnostic and therapeutic approaches to cutaneous disease. Semin. Cutan. Med. Surg..

[23] Sharma D, Kober MM, Bowe WP (2016). Anti-Aging Effects of Probiotics. J. Drugs Dermatol..

[24] Tagami H (2007). Scientific characterization of subclinical skin changes by noninvasive biophysical methods for development of more efficacious skincare products. Int. J. Cosmet. Sci..

[25] Li X, Galzote C, Yan X, Li L, Wang X (2014). Characterization of Chinese body skin through *in vivo* instrument assessments, visual evaluations, and questionnaire: influences of body area, inter-generation, season, sex, and skin care habits. Skin Res. Technol..

[26] Ling Z (2013). Pyrosequencing analysis of the human microbiota of healthy Chinese undergraduates. BMC Genomics.

[27] Xu Z (2016). Dandruff is associated with the conjoined interactions between host and microorganisms. Sci. Rep..

[28] Shanghai Municipal Tourism Administration. Relative Humidity in Shanghai, China: General Information http://www.meet-in-shanghai.net/travel-tips/general-information (2015).

[29] Fox LT (2014). *In Vivo* skin hydration and anti-erythema effects of Aloe vera, Aloe ferox and Aloe marlothii gel materials after single and multiple applications. Pharmacogn. Mag..

[30] Giberti C, Gallo F, Cortese P, Schenone M (2013). Combined intravesical sodium hyaluronate/chondroitin sulfate therapy for interstitial cystitis/bladder pain syndrome: a prospective study. Ther. Adv. Urol..

[31] Costello EK (2009). Bacterial community variation in human body habitats across space and time. Science.

[32] Tanaka A (2014). Molecular characterization of the skin fungal microbiota in patients with seborrheic dermatitis. J. Clin. Exp. Dermatol. Res..

[33] Anderson MA, Whitlock JE, Harwood VJ (2006). Diversity and distribution of Escherichia coli genotypes and antibiotic resistance phenotypes in feces of humans, cattle, and horses. Appl. Environ. Microbiol..

[34] McMurray CL, Hardy KJ, Calus ST, Loman NJ, Hawkey PM (2016). Staphylococcal species heterogeneity in the nasal microbiome following antibiotic prophylaxis revealed by tuf gene deep sequencing. Microbiome.

[35] Moissl-Eichinger C (2017). Human age and skin physiology shape diversity and abundance of Archaea on skin. Sci. Rep..

[36] Capone KA, Dowd SE, Stamatas GN, Nikolovski J (2011). Diversity of the human skin microbiome early in life. J. Invest. Dermatol..

[37] Faust K (2012). Microbial co-occurrence relationships in the human microbiome. PLoS Comput. Biol..

[38] Rosenthal M, Goldberg D, Aiello A, Larson E, Foxman B (2011). Skin microbiota: microbial community structure and its potential association with health and disease. Infect. Genet. Evol..

[39] Leeming JP, Holland KT, Cunliffe WJ (1984). The microbial ecology of pilosebaceous units isolated from human skin. J. Gen. Microbiol..

[40] Meyer K (2015). Evaluation of Seasonal Changes in Facial Skin With and Without Acne. J. Drugs Dermatol..

[41] Galzote C (2014). Characterization of facial skin of various Asian populations through visual and non-invasive instrumental evaluations: influence of seasons. Skin Res. Technol..

[42] Yamamoto A, Takenouchi K, Ito M (1995). Impaired water barrier function in acne vulgaris. Arch. Dermatol. Res..

[43] Jinnestal CL, Belfrage E, Back O, Schmidtchen A, Sonesson A (2014). Skin barrier impairment correlates with cutaneous Staphylococcus aureus colonization and sensitization to skin-associated microbial antigens in adult patients with atopic dermatitis. Int. J. Dermatol..

[44] Lee DE (2015). Clinical Evidence of Effects of Lactobacillus plantarum HY7714 on Skin Aging: A Randomized, Double Blind, Placebo-Controlled Study. J. Microbiol. Biotechnol..

[45] SanMiguel A, Grice EA (2015). Interactions between host factors and the skin microbiome. Cell. Mol. Life Sci..

[46] Braff MH, Bardan A, Nizet V, Gallo RL (2005). Cutaneous defense mechanisms by antimicrobial peptides. J. Invest. Dermatol..

[47] Bahar AA, Ren D (2013). Antimicrobial peptides. Pharmaceuticals (Basel).

[48] Lee SE (2010). Protease-activated receptor-2 mediates the expression of inflammatory cytokines, antimicrobial peptides, and matrix metalloproteinases in keratinocytes in response to Propionibacterium acnes. Arch. Dermatol. Res..

[49] Dinulos JG, Mentele L, Fredericks LP, Dale BA, Darmstadt GL (2003). Keratinocyte expression of human beta defensin 2 following bacterial infection: role in cutaneous host defense. Clin. Diagn. Lab. Immunol..

[50] Wanke I (2011). Skin commensals amplify the innate immune response to pathogens by activation of distinct signaling pathways. J. Invest. Dermatol..

[51] Zaniboni MC, Samorano LP, Orfali RL, Aoki V (2016). Skin barrier in atopic dermatitis: beyond filaggrin. An. Bras. Dermatol..

[52] Kikuchi K (2003). Improvement of mild inflammatory changes of the facial skin induced by winter environment with daily applications of a moisturizing cream. A half-side test of biophysical skin parameters, cytokine expression pattern and the formation of cornified envelope. Dermatology.

[53] Grice EA (2015). The intersection of microbiome and host at the skin interface: genomic- and metagenomic-based insights. Genome Res..

