# Supplementary material for: Topographical diversity of common skin microflora and its association with skin environment type: An observational study in Chinese women
Source: Sci Rep. 2017 Dec 22;7:18046. doi: 10.1038/s41598-017-18181-5 (PMC5741767; doi:10.1038/s41598-017-18181-5)
Supplement: Supplementary file 1 — Supplementary information [file 41598_2017_18181_MOESM1_ESM.doc]

**Topographical diversity of common skin microflora and its association with skin environment type: An observational study in Chinese women**

Xi Li^1*^, Chao Yuan^2*^, Licong Xing^1^, Philippe Humbert^3.4^

^1^ Clinical Research APAC, Johnson & Johnson (China) Ltd., Worldwide EM Innovation Center 3285 Dongchuan Road, Minhang District, Shanghai 200245, China

^2^ Skin & Cosmetic Research Department, Shanghai Skin Disease Hospital, 1278 Bao De Road, Jing’an District, Shanghai, China, 200443

^3^ Department of Dermatology, Research and Clinic Centre on the Tegument, Clinical Investigation Center, Besançon University Hospital, Besançon, France

^4^ University of Franche-Comté, Inserm U1098, Besançon, France

^*^These authors had equal contributions.

**Corresponding author:**

Dr. Xi Li

Johnson & Johnson (China) Ltd.,

Worldwide EM Innovation Center,

3285 Dongchuan Road, Minhang District, Shanghai 200245

China.

**Email:** Cli9@its.jnj.com

**Phone:** 021-24168018

**Fax:** 021-64302010

# **Supplementary Content**

Table S1: Specific primers for each microorganism

| **Target germ** | **Primer** | **Sequence (5’–3’)** | **Size (bp)** |
| --- | --- | --- | --- |
| *Lactobacillus spp* | lac-F | AGCAGTAGGGAATCTTCCA | 345 |
|  | lac-R | ATTCCACCGCTACACATG |  |
| *Propionibacterium acnes* | PA-F | GCGTGAGTGACGGTAATGGGTA | 131 |
|  | PA-R | TTCCGACGCGATCAACCA |  |
| *Staphylococcus epidermidis* | Se705-F | ATCAAAAAGTTGGCGAACCTTTTC | 125 |
|  | Se705-R | CAAAAGAGCGTGGAGAAAAGTATC |  |
| *Staphylococcus aureus* | femA-F | AACTGTTGGCCACTATGAGT | 306 |
|  | femA-R | CCAGCATTACCTGTAATCTCG |  |
| Pseudomonadaceae | PsI-F | GGTGGGCACTCTAAGGAGAC | 173 |
|  | PsI-R | TGCGATCCGGACTACGAT |  |
| *Malassezia furfur* | Mala-F | CTCGCGTACAACGTCTCTGG | 226 |
|  | Mala-R | CGCTGCGTTCTTCATCGA |  |

F, forward primer; R, reverse primer.

**Table S2: A) Co-occurrence rate in different skin types, B) Cochran-Mantel-Haenszel test of co-occurrence rate between different skin types**

| **Microorganism 1** | **Microorganism 2** | **Co-occurrence Rate (%)** | | | | |
| --- | --- | --- | --- | --- | --- | --- |
|  |  | **Dry** | **Normal** | **Oily** | **Exposed** | **Unexposed** |
| *S. aureus* | *S. epidermidis* | 17 | 38 | 19 | 19.333 | 43.333 |
| *S. aureus* | Lactobacillus group | 52 | 77.25 | 67 | 59.667 | 83 |
| *S. aureus* | *M. furfur* | 71 | 85 | 84 | 78.667 | 86.333 |
| *S. aureus* | *P. acne* | 31 | 44.5 | 19 | 30.333 | 45.667 |
| *S. aureus* | Pseudomonadaceae | 16 | 33.25 | 28 | 24.333 | 34.667 |
| *S. epidermidis* | Lactobacillus group | 47 | 49.75 | 42 | 47 | 49 |
| *S. epidermidis* | *M. furfur* | 32 | 44.5 | 29 | 30.667 | 49 |
| *S. epidermidis* | *P. acne* | 66 | 62 | 78 | 66.333 | 64.333 |
| *S. epidermidis* | Pseudomonadaceae | 83 | 62.25 | 75 | 76.333 | 59.333 |
| Lactobacillus group | *M. furfur* | 53 | 73.75 | 67 | 59.667 | 78.667 |
| Lactobacillus group | *P. acne* | 63 | 52.25 | 48 | 56 | 50.667 |
| Lactobacillus group | Pseudomonadaceae | 56 | 46.5 | 51 | 53.333 | 44.333 |
| *M. furfur* | *P. acne* | 52 | 49 | 25 | 42.333 | 48.667 |
| *M. furfur* | Pseudomonadaceae | 39 | 43.75 | 36 | 39 | 44.333 |
| *P. acne* | Pseudomonadaceae | 69 | 60.25 | 67 | 66 | 59.667 |

B.

|  | **Microorganism 1** | **Microorganism 2** | **Exposed or unexposed^#^** | **Normal/ dry/ oily^##^** |
| --- | --- | --- | --- | --- |
| 1 | *S. aureus* | *S. epidermidis* | 0.044189931 | 0.037074426 |
| 2 | *S. aureus* | Lactobacillus group | 0.044797969 | 0.027456594 |
| 3 | *S. aureus* | *M. furfur* | 0.21808716 | 0.181117488 |
| 4 | *S. aureus* | *P. acne* | 0.155748165 | 0.1579784 |
| 5 | *S. aureus* | Pseudomonadaceae | 0.960055089 | 0.976203623 |
| 6 | *S. epidermidis* | Lactobacillus group | 0.02083548 | 0.001772152 |
| 7 | *S. epidermidis* | *M. furfur* | 0.031496964 | 0.016813676 |
| 8 | *S. epidermidis* | *P. acne* | 1.91E-05 | 1.40E-05 |
| 9 | *S. epidermidis* | Pseudomonadaceae | 2.70E-05 | 1.11E-05 |
| 10 | Lactobacillus group | *M. furfur* | 0.00248388 | 0.001285599 |
| 11 | Lactobacillus group | *P. acne* | 3.04E-05 | 6.06E-06 |
| 12 | Lactobacillus group | Pseudomonadaceae | 0.000112592 | 6.49E-05 |
| 13 | *M. furfur* | *P. acne* | 0.005764262 | 0.002936236 |
| 14 | *M. furfur* | Pseudomonadaceae | 4.81E-05 | 5.36E-05 |
| 15 | *P. acne* | Pseudomonadaceae | 0.001218705 | 0.000708712 |

^#^Cochran-Mantel-Haenszel test of co-occurrence rate by strata of whether skin site is exposed or unexposed.

^##^Cochran-Mantel-Haenszel test of co-occurrence rate by strata of whether skin site is dry, oily or normal

# **Figure S1: Shannon and Simpson index plot for relative occurrence of microorganism at different skin sites**


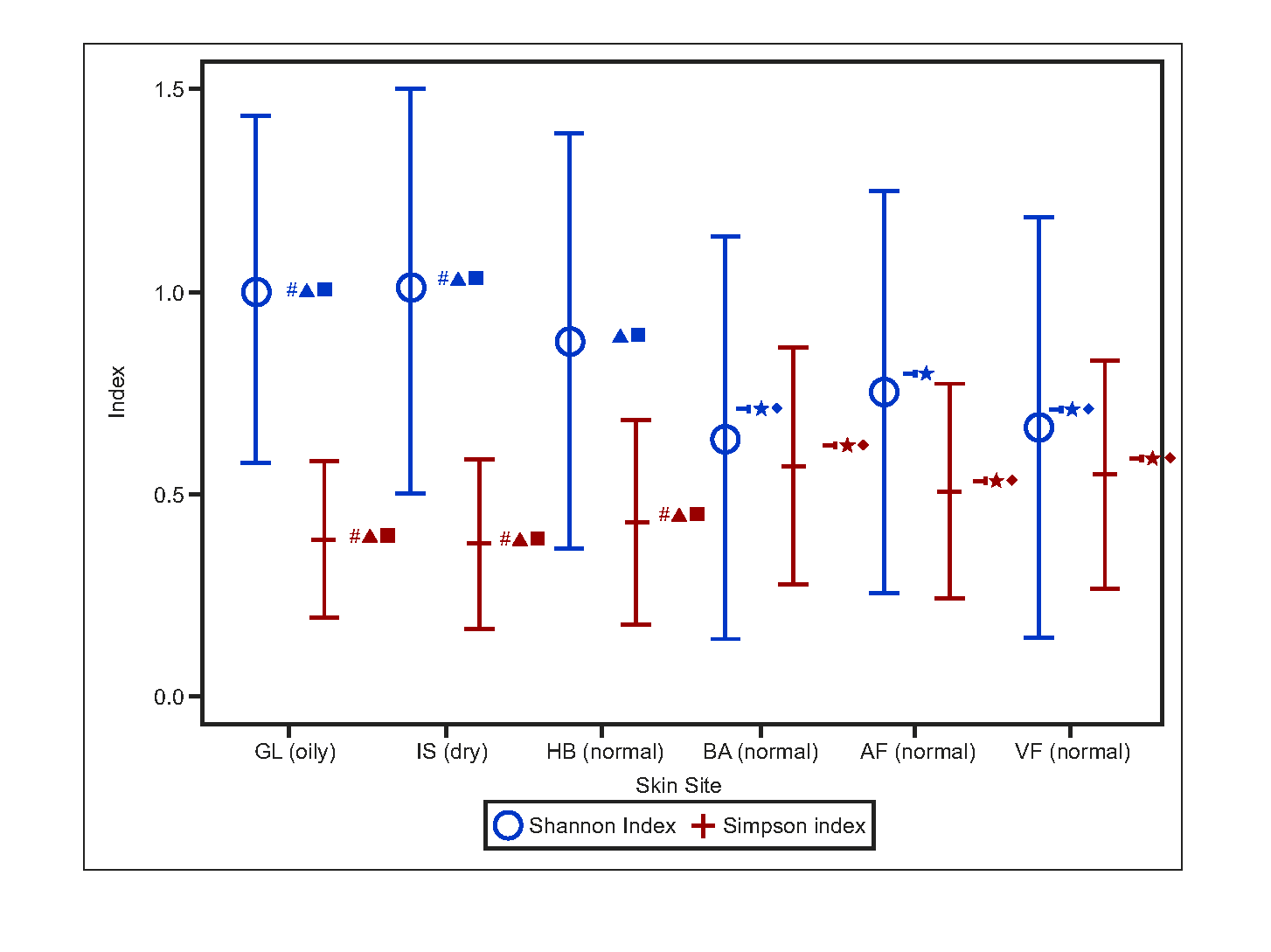


Abbreviations: AF, antecubital fossa; BA, back; GL, glabella; HB, hand-back; IS, interdigital web space; VF, volar forearm.

^╡^,P<0.05,compared to GL; ^★^,P<0.05,compared to IS; ^◆^,P<0.05,compared to HB; ^■^,P<0.05,compared to BA; ^#^,P<0.05,compared to AF; ^▲^,P<0.05,compared to VF
